# A Case of Bing–Neel Syndrome Successfully Treated with Ibrutinib

**DOI:** 10.1155/2018/8573105

**Published:** 2018-08-28

**Authors:** Daniel S. O'Neil, Mark A. Francescone, Karen Khan, Alobeid Bachir, Owen A. O'Connor, Ahmed Sawas

**Affiliations:** ^1^Herbert Irving Comprehensive Cancer Center, Columbia University Medical Center-College of Physicians and Surgeons, New York, NY, USA; ^2^Department of Radiology, Columbia University Medical Center-College of Physicians and Surgeons, New York, NY, USA; ^3^Center for Lymphoid Malignancies, Columbia University Medical Center-College of Physicians and Surgeons, New York, NY, USA; ^4^Department of Pathology and Cell Biology, Columbia University Medical Center-College of Physicians and Surgeons, New York, NY, USA

## Abstract

Bing–Neel syndrome is a rare manifestation of Waldenström macroglobulinemia characterized by lymphoplasmacytic cells' infiltration into the central nervous system. We present a case of a 74-year-old patient with a known diagnosis of Waldenström macroglobulinemia and newly depressed consciousness. Flow cytology of his cerebral spinal fluid demonstrated a lambda light chain-restricted population of B-cells consistent with a CD5+ CD10+ B-cell lymphoma. Magnetic resonance imaging suggested involvement of the left optic nerve sheath and the bilateral orbital and parietal parenchyma and leptomeninges. He was diagnosed with Bing–Neel syndrome and treated with intrathecal liposomal cytarabine, intravenous high-dose methotrexate, and rituximab without improvement. Subsequently, he started treatment with ibrutinib 560 mg daily and concurrent rituximab. Within three months, he showed clinical and radiologic improvement. The patient has continued on ibrutinib and has now been stable for over 36 months. This represents the longest reported period of successful treatment of Bing–Neel syndrome with ibrutinib.

## 1. Introduction

Waldenström macroglobulinemia (WM) is a rare B-cell lymphoma, with an incidence of just 0.3 per 100,000 person-years [1]. While most neurologic complications of WM are neuropathies secondary to IgM activity, direct central nervous system (CNS) infiltration by malignant lymphoplasmacytic cells (LPCs) is a recognized complication known as the Bing–Neel syndrome (BNS).

We present a case of a patient with BNS refractory to chemoimmunotherapy achieving a complete response to ibrutinib and maintenance of response for 3 years.

## 2. Case Presentation

The patient presented in November 2010 at the age of 74 years with several splotchy, hyperpigmented lesions across his forehead. He reported that the lesions had been present for two years but were now growing in size. A skin punch biopsy showed scattered dense lymphocytic nodules and follicles in the dermis composed of large centroblast-like and smaller centrocyte-like cells with the appearance of germinal centers. Follicular B-cells expressed CD10 and Bcl-6, were equivocal for Bcl-2, and lacked MUM1 expression. CD21-positive follicular dendritic cell meshworks were associated with the follicles, and the Ki-67 staining index was 20–30%. Plasma cells were appreciated in the center of a few follicles and the interfollicular areas. Flow cytometry identified a population of B cells (3.6% of nucleated cells) with the following phenotypes: CD19+, CD20+, CD79a+, CD5−, CD10−, FMC-7+ (moderately bright), CD43−, CD103−, surface IgM−, surface IgD−, and CD52+. No IGH/BCL2 translocation was detected in the skin biopsy.

Bone marrow biopsy in December 2010 showed diffuse infiltration of small lymphocytes and mature-appearing plasma cells. CD20 staining identified B cells (CD23+/−, bcl2+, CD5−, CD10−, and CD43−) accounting for >50% of total cellularity, and CD138 showed lambda-restricted plasma cells (IgM+) accounting for 20–30% of cellularity. Serum protein electrophoresis performed at the same time detected a monoclonal IgM lambda protein at 2.6 g/dL concentration. Positron emission tomography-computed tomography (PET-CT) imaging showed hypermetabolic left axillary lymphadenopathy and osseous lesions within the T5 vertebral body and left iliac wing; splenomegaly and right axillary lymphadenopathy were also appreciated. At the time of bone marrow biopsy, the patient was anemic, with a hemoglobin of 6.0 g/dL and a mean corpuscular volume of 92.8 fL, and leukopenic, with a white blood cell count of 3.2 × 103/*μ*L and an absolute neutrophil count of 2.2 × 103/*μ*L. Lactate dehydrogenase was elevated at 264 U/L. Laboratory testing of renal and liver function was normal. The diagnosis of WM was made.

Treatment was planned with rituximab and fludarabine for six cycles. The patient completed five cycles with the planned sixth cycle held after he developed a pneumonia.

His disease was quiescent until April 2014, when he presented back to clinic with a mass adjacent to the right brachial plexus causing upper extremity weakness, swelling, and pain. Cytology from fine-needle aspiration of the mass demonstrated clonal intermediate and large lymphoid cells with plasmacytoid features. A clonal IgH rearrangement was present, and the predominant B-cell phenotype on flow cytometry was CD19+, CD79a+, CD20+, CD10+, CD5+, CD23−, CD38+/−, FMC7−, CD43−/+, CD25+, CD103−, HLA-DR+, surface IgM+, and surface IgD+. Molecular testing of the soft tissue sample detected an *MYD88* L265P mutation. Bone marrow biopsy only showed a tiny population (<0.5%) of atypical plasma cells without a detectable clonal IgH rearrangement. He received a single cycle of rituximab-bendamustine combination therapy at an outside institution.

Unfortunately, over the course of July 2014, he developed progressive, intermittent confusion. Brain imaging was difficult to obtain, but cerebrospinal fluid (CSF) contained 420 nucleated cells/*µ*L and protein at 296 mg/dL. CSF cytology was highly cellular with cells cytomorphologically consistent with non-Hodgkin's lymphoma, and flow cytometry showed a predominant population of lambda light chain-restricted B-cell phenotype CD19+, CD79a+, CD20+, CD10+, CD5+, CD23−, CD38+/1, FMC7−, CD43+, CD103−, surface IgM+, and surface IgD+. Again, an *MYD88* L265P mutation was detected. Serum IgM was 942 mg/dL; monoclonal protein concentration was 0.8 g/dL. He was treated with intrathecal liposomal cytarabine, intravenous high-dose methotrexate, and rituximab 750 mg every 4 weeks. His mental status improved initially, but he subsequently developed bacterial meningitis. His meningitis led to a prolonged hospitalization complicated by recurrent fevers of idiopathic etiology, aspiration pneumonia, balanitis, *Clostridium difficile* colitis, and declining mental status. Treatment was continued as permitted by recurrent infections. By October 2010, CSF studies still showed few lymphocytes suspicious for refractory disease, and the patient's mental status remained poor.

**Figure 1:**
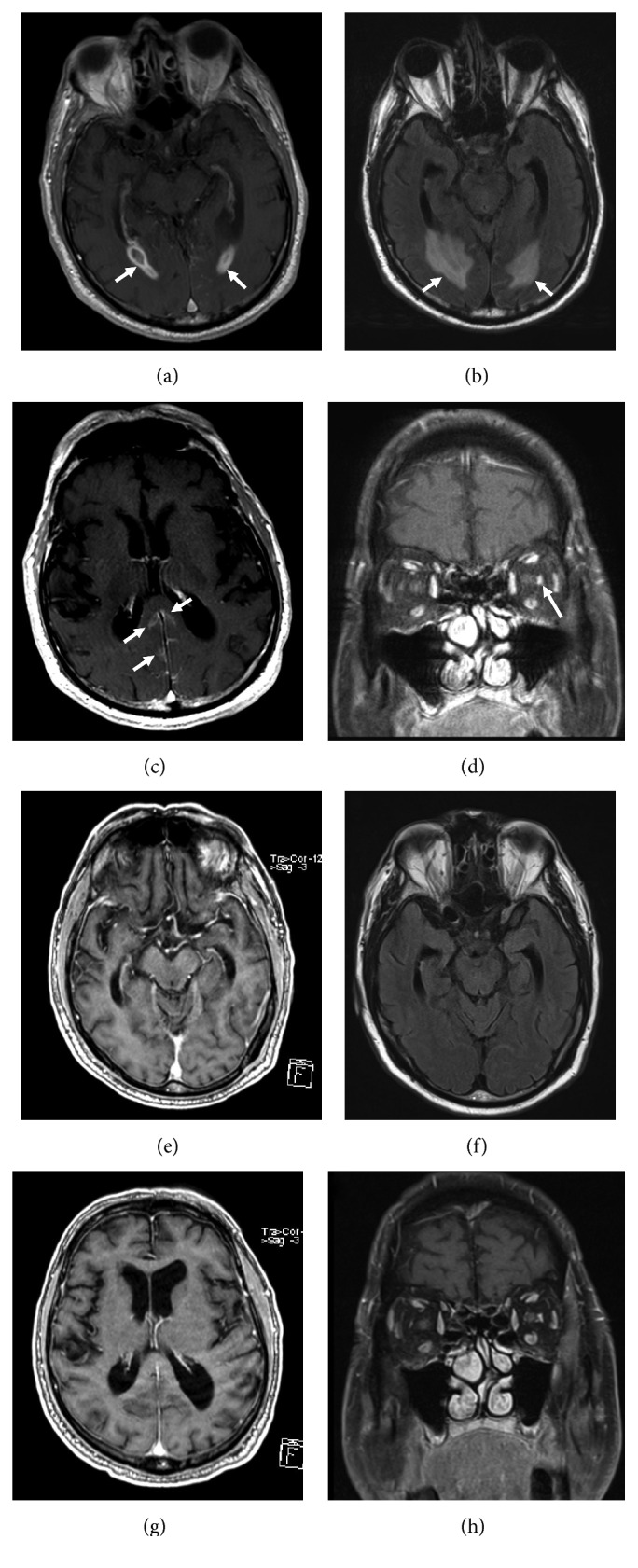
Magnetic resonance imaging of patient's brain obtained before and after initiation of ibrutinib. (a) Subependymal enhancement in December 2014 (T1 postcontrast). (b) Periventricular hyperintensity in December 2014 (FLAIR). (c) Leptomeningeal enhancement in December 2014 (postcontrast). (d) Left optic sheath enhancement in January 2015 (postcontrast). (e) Subependymal enhancement resolved in March 2015 (T1 postcontrast). (f) Periventricular hyperintensity resolved in March 2015 (FLAIR). (g) Leptomeningeal enhancement resolved in March 2015 (postcontrast). (h) Left optic sheath enhancement decreased in March 2015 (postcontrast).

Magnetic resonance imaging (MRI) of the brain and orbits from December 2014 showed subcutaneous ependymal enhancement within the atria of both lateral ventricles with extensive bilateral adjacent periventricular parenchymal FLAIR hyperintensity, evidence of leptomeningeal spread in the parasagittal portion of both occipital and parietal lobes, and enhancement of the bilateral optic nerves and the left optic nerve sheath concerning for lymphomatous involvement (Figure 1).

In the late December 2014, our patient was started on ibrutinib 560 mg daily and continued rituximab. By February 2015, his mental status was clear, and he was reporting improvements in his vision and hearing. MRI brain was repeated in March 2015, showing interval improvement with decreased signal abnormality adjoining the atrium of the lateral ventricle and only mild persistent left optic nerve sheath enhancement (Figure 1). By that time, serum IgM was 738 mg/dL and monoclonal protein was 0.5 g/dL. By July 2015, MRI showed no abnormal enhancement in the brain at all. He has continued ibrutinib and periodic rituximab infusion through December 2016 with persistently normal MRI. Treatment has been complicated by hypogammaglobulinemia requiring occasional intravenous immunoglobulin administration and a hospital admission for methicillin-sensitive *Staphylococcus aureus* bacteremia. Otherwise, as of March 2018, he is doing well with minimal cytopenias or adverse effects.

## 3. Discussion

The clinical manifestations of BNS are diverse and, often, nonspecific. Patients may present with focal complaints, such as visual and auditory defects, or global manifestations, such as seizures or reduced consciousness. Per recently published expert consensus guidelines, the diagnosis is confirmed with biopsy of the involved parenchyma or leptomeninges and demonstration of LPC infiltration. In the absence of CNS biopsy, CSF can be examined for the presence of LPCs via cytology, flow cytometry, and molecular testing for clonal IgG gene rearrangement and *MYD88* L265P mutations. Immunofixation and electrophoresis may also identify IgM monoclonal proteins in the CSF [2]. Magnetic resonance imaging enhancement may be diffuse or localized. At the time of initial presentation, our patient met the Second International Workshop on Waldenstrom's Macroglobulinemia clinical criteria for the diagnosis of WM; the value of *MYD88* in the diagnosis of WM was not well described until 2012 [3, 4]. Soft tissue samples and CSF from 2014 did show *MYD88* L265P mutations, and the development of a CD5+ CD10+ phenotype does not exclude WM [5].

BNS is usually diagnosed in patients already known to have WM, but it has been reported as the first clinical manifestation of WM in 15% to 36% of cases [2]. A case series of 34 patients suggest that those with BNS as the initial presentation of WM have a better prognosis than those with a secondary presentation [6]. That same series report a median overall survival of just 59% at 3 years following diagnosis with BNS. While therapy typically includes intrathecal chemotherapy, high-dose methotrexate, systemic chemotherapy with fludarabine or bendamustine, or immunotherapy with rituximab, only rituximab use is associated with improved survival [6].

Ibrutinib is a potent inhibitor of Bruton's tyrosine kinase, a B-cell receptor involved in several signaling pathways essential to B-cell development and survival [7]. In previously treated patients with WM, ibrutinib monotherapy has an excellent overall response rate, exceeding 90%. Mutations to *MYD88* or *CXCR4* seem to improve the likelihood of response [8].

Ibrutinib is presently granted US Federal Drug Administration approval for use in chronic lymphocytic leukemia (CLL), mantle cell leukemia (MCL), marginal zone lymphoma, chronic graft-versus-host disease, and WM [9]. Activity against central nervous system lesions has been described in several of these diagnoses. Six patients in a series of 30 CLL patients with CNS disease confirmed by cerebral spinal fluid (CSF) analysis were treated with ibrutinib; four patients had received at least 2 prior lines of therapy. All patients showed some response, with three complete responses, and five were still alive at a median follow-up of 8 months [10]. For MCL, a small case series of three patients with intracerebral lesions all demonstrated clinical examination improvement within 3 to 8 days of initiating single-agent ibrutinib at the standard dose. Two of three showed complete response by MRI and PET-CT, with ongoing response at 1 year and 9 months of follow-up [11]. Similar results were reported among five MCL patients from the United Kingdom. All five patients achieved a response in CNS disease with ibrutinib, with a median length of 4 months (range 4 days–5 months), although two patients received concurrent systemic chemotherapy [12].

Individual case reports describe five patients with WM and CNS involvement undergoing treatment with ibrutinib, either as first-line therapy or after progressing on rituximab and systemic and intrathecal chemotherapy (Table 1). Response was reported for four, with the length of measured response lasting from 6 to 23 months [13–16]. CSF concentrations of ibrutinib were measured in several of these cases. In two MCL patients, they were 4.5 and 113.5 nmol/L, and one WM patient peaked at 34 nmol/L [11, 14]. These concentrations demonstrate ibrutinib's capacity to cross the blood-brain barrier and were all greater than its half maximal inhibitory concentration (0.5 nmol/L).

This case represents another instance of rapid, dramatic, and durable improvement of BNS upon initiation of ibrutinib. His response has now been maintained for over 36 months, the longest yet reported. The multiple instances of durable improvement of CNS infiltrates in CLL and MCL with ibrutinib and the growing number of similar responses in BNS suggest that ibrutinib should be routinely considered among the therapeutic options for these challenging patients.

## Figures and Tables

**Table 1:** Summary of previously reported cases of ibrutinib use for the treatment of Bing–Neel syndrome.

Case report	Number of patients	Prior diagnosis of WM	Earlier BNS therapies	Ibrutinib dose	Length of response to ibrutinib
Cabannes-Hamy et al. [13]	2	Yes, yes	R/HD-MTX/L-AC → HDAC, R-DHAC → R	420 mg daily, 420 mg daily	6 months, 6 months
Mason et al. [14]	1	Yes	R/HD-MTX → B → L-AC	560 mg daily	23 months
Varettoni et al. [15]	1	No	BR/IT-MTX	Not reported	Not reported
Boudin et al. [16]	1	No	None	420 mg daily	11 months

BNS = Bing–Neel syndrome; WM = Waldenström macroglobulinemia; R = rituximab; HD-MTX = high-dose methotrexate; L-AC = intrathecal liposomal cytarabine; HDAC = high-dose cytarabine; R-DHAC = rituximab, dexamethasone, high-dose cytarabine, and carboplatin; B = bendamustine; BR = bendamustine and rituximab; IT-MTX = intrathecal methotrexate.

## Data Availability

The individual patient clinical data used to report this case study have not been made available because they contain protected health information.
