# Erratum to “A Case of Bing–Neel Syndrome Successfully Treated with Ibrutinib”

**DOI:** 10.1155/2018/7610201

**Published:** 2018-10-29

**Authors:** Daniel S. O'Neil, Mark A. Francescone, Karen Khan, Bachir Alobeid, Owen A. O'Connor, Ahmed Sawas

**Affiliations:** ^1^Herbert Irving Comprehensive Cancer Center, Columbia University Medical Center, College of Physicians and Surgeons, New York, NY, USA; ^2^Department of Radiology, Columbia University Medical Center, College of Physicians and Surgeons, New York, NY, USA; ^3^Center for Lymphoid Malignancies, Columbia University Medical Center, College of Physicians and Surgeons, New York, NY, USA; ^4^Department of Pathology and Cell Biology, Columbia University Medical Center, College of Physicians and Surgeons, New York, NY, USA

In the article titled “A Case of Bing–Neel Syndrome Successfully Treated with Ibrutinib” [1], the name format of the fourth author was given incorrectly as Alobeid Bachir. It should have been written as Bachir Alobeid. The corrected author list is shown above.
